# Patient satisfaction with HIV and TB treatment in a public programme in rural KwaZulu-Natal: evidence from patient-exit interviews

**DOI:** 10.1186/1472-6963-14-32

**Published:** 2014-01-23

**Authors:** Natsayi Chimbindi, Till Bärnighausen, Marie-Louise Newell

**Affiliations:** 1 Wellcome Trust Africa Centre for Health and Population Sciences, University of KwaZulu-Natal, Durban, South Africa; 2 School of Public Health, University of the Witwatersrand, Johannesburg, South Africa; 3 Department of Global Health and Population, Harvard School of Public Health, Boston, USA; 4 Faculty of Medicine and Faculty of Human and Social Sciences, University of Southampton, Southampton, UK

**Keywords:** Patient satisfaction, Factor analysis, HIV, TB, Health systems

## Abstract

**Background:**

Patient satisfaction is a determinant of treatment uptake, adherence and retention, and an important health systems outcome. Queues, health worker-patient contact time, staff attitudes, and facility cleanliness may affect patient satisfaction. We quantified dimensions of patient satisfaction among HIV and TB patients in a rural sub-district of KwaZulu-Natal, South Africa, and identified underlying satisfaction factors that explained the data.

**Methods:**

We conducted patient-exit interviews with 300 HIV and 300 TB patients who were randomly selected using a two-stage cluster random sampling approach with primary sampling units (primary healthcare clinics) selected with probability-proportional-to-size sampling. We performed factor analysis to investigate underlying patient satisfaction factors. We compared the satisfaction with HIV and TB services and examined the relationships between patient satisfaction and patients’ socio-demographic characteristics in multivariable regression.

**Results:**

Almost all patients (95% HIV, 97% TB) reported to be globally satisfied with the healthcare services received on the day of the interview. However, patient satisfaction with specific concrete aspects of the health services was substantially lower: 52% of HIV and 40% of TB patients agreed that some staff did not treat patients with sufficient respect (p = 0.02 for difference between the two patient groups); 65% of HIV and 40% of TB patients agreed that health worker queues were too long (p < 0.001). Based on factor analysis, we identified five factors underlying the HIV data and the TB data (availability, accommodation, acceptability and communication for HIV and TB patients; health worker preference for HIV patients only; and global satisfaction for TB patients only). The level of satisfaction did not vary significantly with patients’ socio-demographic characteristics.

**Conclusions:**

In this rural area, HIV and TB patients’ evaluations of specific aspects of health services delivery revealed substantial dissatisfaction hidden in the global assessments of satisfaction. A wide range of patient satisfaction variables could be reduced to a few underlying factors that align broadly with concepts previously identified in the literature as affecting access to healthcare. Increases in health systems resources for HIV and TB, but also improvements in facility maintenance, staff attitudes and communication, are likely to substantially improve HIV and TB patients’ satisfaction with the care they receive in public-sector treatment programmes in rural communities in South Africa.

## Background

The epidemics of HIV and TB in sub-Saharan Africa are closely related and particularly persistent. Currently, approximately 33 million people are HIV infected globally, with South Africa having the greatest absolute number of HIV-infected individuals in the world [1]. Globally, the World Health Organization (WHO) estimates that 8.7million new cases of TB were reported in 2011 (13% of these being co-infected with HIV) [2]. South Africa contributes a substantial proportion of the global burden of TB, for example in 2010, 490 000 cases of TB were recorded in the country [3,4]. In rural Hlabisa sub-district of KwaZulu-Natal, both HIV and TB remain major causes of morbidity and mortality, despite the recent impact of ART on mortality and HIV incidence [5-9]. HIV prevalence among resident adults in 2010 was 29% and TB prevalence was almost 25% among those initiated on ART in 2006 [10,11]. While TB treatment has been widely available in this area for more than three decades [12], ART only became available in public services in South Africa in 2004 [13]. Since then, the Hlabisa HIV Treatment and Care Programme (ART programme) has provided HIV treatment and care at an increasing number of primary healthcare (PHC) clinics (16 at the time of the study). By 2011, 37% of all HIV infected people in the study area had been initiated on ART [6].

Patient satisfaction is an important outcome of health systems [14]. It can be defined as the perceived fulfillment of patients’ needs and desires through the delivery of healthcare [15,16]. Patient satisfaction with HIV and TB treatment is important for two main reasons. First, it is an important outcome in its own right as a health systems goal. Many of the well-known frameworks to structure health systems thinking, such as WHO’s building blocks [17] and the “control knobs” framework [14], include a measure of patients’ subjective evaluation of health services, such as “patient satisfaction” [14] or “responsiveness” [17] as one of the main health systems outcomes. Second, patients who are satisfied with the healthcare received in the healthcare facility were less likely to face barriers to access and challenges to treatment adherence [18,19]. Quantifying and understanding HIV and TB patients’ satisfaction with public-sector treatment programmes is thus important for developing strategies to ensure that both health systems goals are attained. Understanding the level, dimensions and determinants of patient satisfaction is particularly topical in the South African context, for informing the current efforts at reforming the national health system [20].

While in Hlabisa sub-district in recent years HIV and TB services have been increasingly integrated at the level of front-line delivery [21,22], HIV and TB treatment and care services are still largely vertically administered regarding planning, funding, and monitoring and evaluation, despite the highly overlapping patient clientele. The two programmes have very different histories and lengths of operation; the TB Directly Observed Treatment Short-Course (DOTS) strategy was introduced in KwaZulu-Natal in 1996 through the National TB control programme to improve cure rates and reduce drug resistance [12,21,23], while HIV treatment only became available through the public-sector health system in KwaZulu-Natal in 2004 and has since then been progressively scaled up [13,22,24-27]. It is thus plausible that patient satisfaction differs substantially across the two programmes. The TB programme has had more than three decades to learn how to best accommodate and respond to patients’ demands. On the other hand, the HIV programme is relatively young and structures and processes are likely to be less well-adapted to patients’ needs and desires.

In this paper we quantified dimensions of patient satisfaction among HIV and TB patients attending public-sector PHC clinics in rural KwaZulu-Natal. We further identified underlying satisfaction factors that explained the data, compared satisfaction between HIV and TB patients and determined the extent to which socio-demographic patient characteristics are related to the different patient satisfaction factors.

## Methods

### Study area

Hlabisa sub-district located in the uMkhanyakude district in northern KwaZulu-Natal is predominately rural with a population of approximately 228 000. The Hlabisa HIV Treatment and Care Programme is a Department of Health (DoH) programme which has received operational support from the Wellcome Trust Africa Centre for Health and Population Sciences, (Africa Centre) and financial support from the Presidential Emergency Fund for AIDS Relief (PEPFAR) [27]. (http://www.africacentre.com) The DoH TB programme does not directly receive external donor support, but the externally-supported HIV treatment programme has supported the integration of the TB and the HIV treatment programmes. Both programmes utilize a decentralized model of healthcare delivery and were available at all 16 PHC clinics at the time of this study [27], but HIV and TB treatment services are delivered by different front-line health workers [21,22].

The HIV and TB treatment programmes operate according to the South African DoH guidelines [12,27,28]. All the PHC clinics in the sub-district offer HIV counseling and testing (both provider-initiated and through standard voluntary counseling and testing (VCT) centres) [27,29,30]. HIV patients visit the clinic monthly in the first year of treatment and two-monthly thereafter if they are stable on treatment, for counselling, assessment and drug collection. TB nurses identify TB suspects at each PHC clinic and collect sputum which is sent to the Hlabisa National Health Laboratory Services (NHLS) for acid-fast bacilli (AFS) smear testing. All smear-positive patients are initiated onto first-line standard TB regimens and patients with negative smear who remain symptomatic are referred to the district hospital for further assessment [11,21]. TB patients visit the clinic once every month for review and to collect their TB medication; patients with multi-drug resistant (MDR) and extensively-drug resistant (XDR) TB are hospitalized at the district hospital for one to two months with further follow-up at the PHC clinic. TB treatment is six months for routine TB cases and up to 24 months for MDR and XDR patients.

### Data sources and sampling

In 2009 we collected data in a survey that was part of a larger multi-site study called Researching Equity in ACcess to Health Care (REACH) [31,32]. For the study presented here, we used data from patient-exit interviews that were part of the REACH study to examine patient satisfaction with different aspects of treatment delivery among patients utilizing HIV and TB treatment and care services. The sample size for the final sampling unit (300 HIV and 300 TB patients) was established through a formal power calculation to ensure a sufficiently large sample to detect significant differences in several key indicators, including patient satisfaction, while accounting for the expected clustering of indicator values at the level of the PHC clinics where we approached patients for the interviews [33]. We used a two-stage cluster random sampling approach, first selecting a random sample of PHC clinics within the sub-district (with replacement) and then randomly sampling the same number of patients (60) in each facility in the second sampling stage [34]. The number of patients (i.e., the second-stage sampling units) was determined in the power calculation, given the number of clinics we decided to visit based on operational feasibility of this research work.

In the first sampling stage, we randomly drew five PHC clinics with probability-proportional-to-size – i.e., larger facilities had a larger probability of being selected into the sample. Because we sampled with replacement, it was possible for one clinic to be selected multiple times. The initial first-stage sampling frame comprised of the 16 PHC clinics in the district, which delivered both HIV and TB treatment. In three clinics other research projects were ongoing and these three clinics were removed from the initial first-stage sampling frame to avoid participant fatigue and over-researching; furthermore, the four clinics which had a patient load of fewer than 60 ART or 60 TB patients were excluded, leaving a final first-stage sampling frame of nine clinics. According to the HIV treatment programme statistics, the number of patients per clinic who were on ART by 2008 in the 16 PHC clinics in the initial sampling frame, ranged from 34 to 1006, and the number of patients on ART who were in the four clinics that were selected in the first sampling stage was 213, 260, 381 and 635 respectively. Based on the DoH statistics for Hlabisa sub-district, by 2008 the number of TB patients in the 16 PHC clinics ranged from 12 to 250, and the number of TB patients who were in the five clinics selected in the first sampling stage was 100, 100, 133, 160 and 250. Four trained fieldworkers conducted the patient-exit interviews using the local language in the study area, *isiZulu*, with 60 patients randomly selected from three clinics each and 120 patients from one clinic that was sampled twice (HIV patients) and with 60 patients selected from each of five clinics (TB patients). We received ethical clearance for the study from University of KwaZulu-Natal (BE174/08).

**Figure 1:**
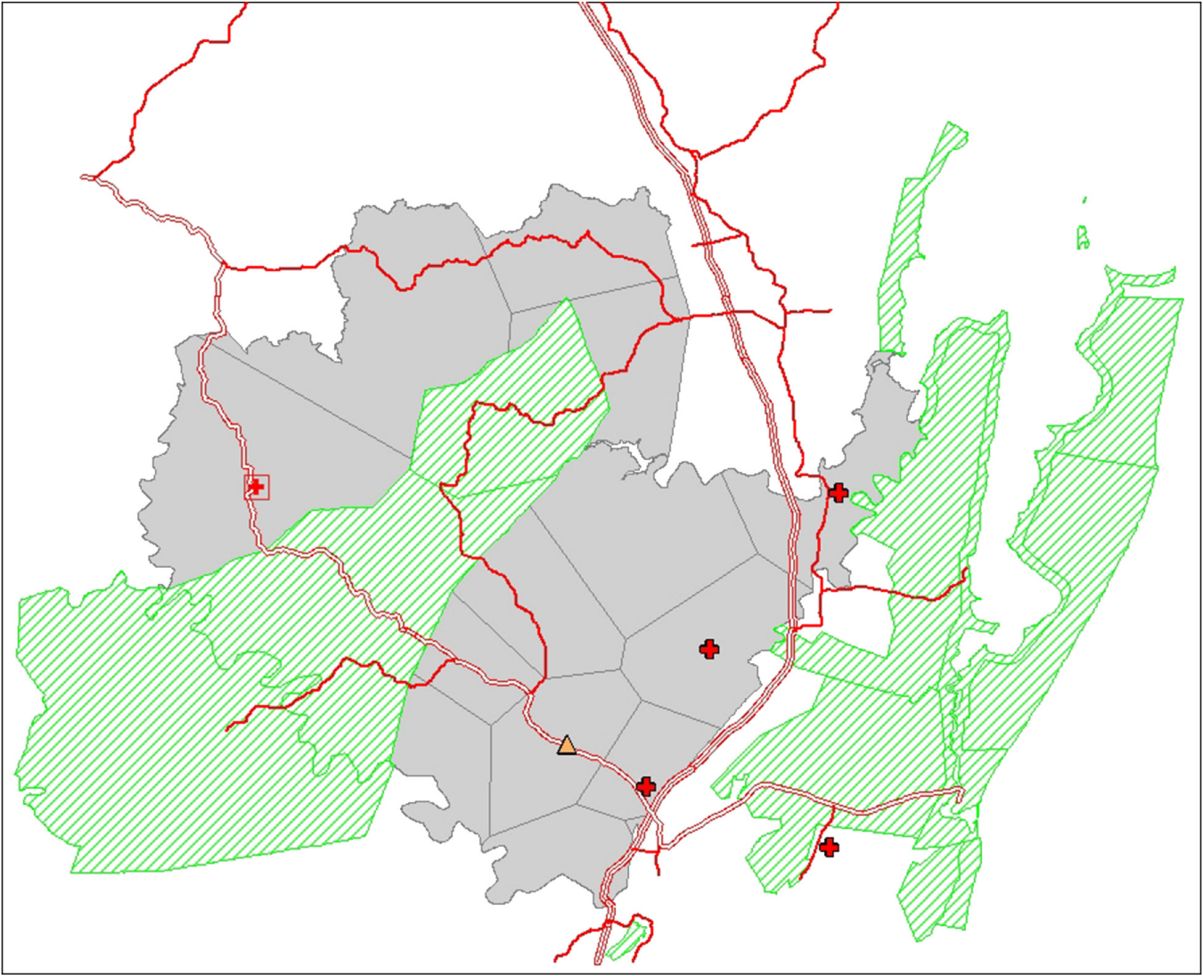
**Map of Hlabisa sub-district.** Figure 1 shows a map of Hlabisa sub-district (in grey) and the location of the clinics in which the study was conducted. The primary healthcare (PHC) clinics are shown as red crosses; the square with a red cross indicates the location of the PHC clinic located on the premises of the district hospital. The Africa Centre is shown as a yellow triangle.

Figure 1 shows a map of Hlabisa sub-district (in grey) and the location of the clinics in which the study was conducted. The primary healthcare (PHC) clinics are shown as red crosses; the square with a red cross indicates the location of the PHC clinic located on the premises of the district hospital. The Africa Centre is shown as a yellow triangle. TB patients were interviewed in all of the five clinics shown; ART patients were interviewed in all of the clinics shown with the exception of the clinic that is located lowest on this map.

### Data collection instrument and variables

We used a structured patient-exit interview questionnaire, which included multiple questions on patient satisfaction. The same questionnaire was used for HIV and TB treatment patients [33]. Patient satisfaction is a multidimensional construct, which focuses on patient perceptions and evaluation of the treatment and care they receive [35]. Several questions to collect information on patient satisfaction have been used in multiple studies in sub-Saharan Africa, and have been validated and subjected to reliability analysis. We used such established questions about patient satisfaction for this study to elicit patient satisfaction with the overall (or global) experience during the treatment visit, staff respect, privacy and confidentiality, staff attitudes, communication, staff competency, and physical environment [36-43]. The responses for satisfaction outcomes were categorical (“always”, “sometimes” or “never”; or “agree”, “disagree”, “both agree and disagree”) or binary (“yes” or “no”) (see Table 1). For the analyses (see below), the categorical responses were coded into binary variables (“always” vs. “not always” and “agree” vs. “not agree”). In total we used 13 questions from the multisite study questionnaire (Table 1) for this analysis of patient satisfaction.

**Table 1:** Measures of the indicators of patient satisfaction with healthcare

**Statement/question**	**Response categories**
1. How satisfied were you with the service today?	Very satisfied/satisfied; neither satisfied nor dissatisfied; dissatisfied/very dissatisfied; don’t know
2. The doctors and nurses (health workers) discussed the treatment fully with me	
3. I find it easy to tell the health workers when I have missed taking my tablets	(Statement/question 2-10) Agree; disagree; both agree and disagree; don’t know/not sure
4. It is a problem that health workers do not speak my language	
5. The health workers are too busy to listen to my problems	
6. Some staff do not treat patients with sufficient respect	
7. The health workers I see respect me	
8. Patient information is kept confidential in this clinic	
9. The facilities (including waiting area and toilets) are dirty	
10. The queues to see a doctor or nurse are too long at this facility	
11. In this clinic are you able to talk to the doctors or nurses in private?	Always; sometimes; never
12. For your ARV treatment (TB care) what would you prefer:	Nurse; doctor; indifferent; don’t know
a) To see a nurse in a nearby clinic or	
b) To travel further to see doctor	
13. How do you think the service in this clinic could be improved?	Yes; no
a) Shorter queues	
b) More health workers	
c) Cleaner facilities	
d) Better patient facilities (toilets, waiting room area etc)	
e) Don’t know	
f) Other specify	

Patients who agreed to participate in the study were asked to sign a consent form by the fieldworkers. Consent to participate was obtained from all patients who were at least 18 years of age and, for HIV patients, who had been receiving ART for at least two weeks or for TB patients, who were on TB treatment for at least two months. The treatment duration criterion for HIV patients was used to exclude patients who had just been initiated on ART, because initiation visits are very different from the follow-up visits and we intended to focus on the patient satisfaction with routine visits rather than with ART initiations. Since the first visit following ART initiation occurs two weeks after initiation in this setting (unlike the routine visits thereafter), this inclusion criterion ensures that patients had completed at least two ART visits. We chose this criterion to ensure that the experience represented by the patients in our sample was as wide regarding their time on ART as possible. However, the vast majority of patients in the sample had been on treatment for much longer than two weeks: 99% of the patients in the sample of ART patients had been on ART one month or longer and 95% of patients had been on ART for two months or longer. The treatment duration criterion for TB patients was used, because after two months the intensive phase of treatment for patients with drug-resistant TB, which usually occurs in a hospital, would be completed. The intention here is to restrict the sample to patients who are receiving routine care.

### Analysis

We used standard descriptive statistics to present the findings on different indicators of patient satisfaction. Next, we performed a factor analysis with oblique rotation (oblimin rotation) to identify the latent variables, or factors, that generated the patient satisfaction data [44]. Oblique rotation was used because on theoretical grounds it is likely that different patient satisfaction factors are related to each other; e.g. a factor capturing global satisfaction is likely related to several of the factors capturing more specific satisfaction dimensions. The Kaiser criterion (eigenvalue > 1.00) was used to guide decisions regarding which factors to retain and inspection of the factor loadings was used to determine which aspects of patient satisfaction the identified factors capture [38,45,46]. To assess whether socio-demographic characteristics were associated with different patient satisfaction factors, we regressed the five factors identified in the factor analysis on sex, age, marital status, employment status and education level. All these variables have previously been found to influence patient satisfaction levels [42,47]. We controlled for clustering at the clinic level in the regressions. All analyses were done using STATA IC, version 11. We further extracted responses from the open-ended, qualitative questions to aid the interpretation of the quantitative findings. Responses to the open-ended question *“How do you think the service in this clinic could be improved?”* were coded manually into themes and a thematic analysis of the responses was done by one of the authors (NC) through identifying and analyzing common ideas and patterns in the responses from the data.

## Results

Table 2 shows the socio-demographic profile of patients utilizing HIV and TB treatment. More women (62% HIV and 53% TB) than men utilized treatment. Almost all patients were globally satisfied with the service they received on the day of the interview; Table 3 shows that 95% of HIV patients and 97% of TB patients reported being either very satisfied or satisfied with the service they had just received. However, satisfaction levels were substantially lower for some specific dimensions of quality of care particularly among patients utilizing HIV treatment.

a. Staff-patient communication

The majority of patients (96% TB and all 100% HIV) agreed that health workers discussed treatment fully with them. However, 10% of HIV patients and 5% of TB patients did not find it easy to tell the health worker when they had missed taking tablets. Although nearly all patients disagreed that it was a problem that some health workers do not speak the patients’ language, more than a tenth of the patients (15%) utilizing either HIV or TB treatment agreed that health workers were too busy to listen to their problems anyway.

b. Staff attitudes

Level of dissatisfaction with staff attitudes was greater among HIV than TB patients. A significantly higher proportion of HIV (52%) than TB (40%) patients agreed with the statement that some staff do not treat patients with sufficient respect. However, when asked whether they themselves were treated with respect by their healthcare worker, the majority of patients (93% HIV and 96% TB) agreed that they were treated with respect.

c. Privacy and confidentiality

16% of HIV patients and 11% of TB patients reported that they had either sometimes or never been able to talk in private to their doctors and nurses in their past clinic visits. However, a high proportion of patients (96% HIV and 94% TB) agreed that patient information is kept confidential at the clinic.

d. Staffing and amenities

Nurse-based care for HIV and TB treatment and care was highly acceptable to respondents (100% for HIV and 99% for TB). Slightly more than a fifth (21%) of patients utilizing either HIV or TB care agreed that the facilities (including waiting area and toilets) were dirty. Data from open-ended responses provided some indications of the precise sources of dissatisfaction (lack of a water dispenser and cups to drink, shortage of seats in the waiting area, and lack of shelter from rain and sun in the waiting area, which is outside the clinics).

A significantly higher proportion of HIV (65%) than TB (40%) patients agreed that the queues to see a doctor or nurse were too long. Regarding means of improving services in the clinic, a significantly higher proportion of HIV than TB patients reported shorter queues (57% v 35%); having more health workers (57% v 45%) and having better patient facilities (67% v 54%), respectively, as ways of improving the services at the facilities. One TB patient highlighted that “doctors must always be available” while another suggested that “the staff need to work shifts” as a means of improving services in the clinics.

e. Food provision and patient support

One HIV patient suggested food hand-outs at the clinics because patients have to wait very long to fetch their treatment; another patient suggested patient support in the form of clinic patient groups. Patients in both the HIV and TB programmes emphasized that transport could substantially improve their satisfaction with the treatment experience (“they have to transport us because we are far from the hospital”, or “they need to take us from home with the hospital cars”).

f. Staff efficiency and easy access to medication

A few patients told the interviewers during the open-ended part of the interviews that they hoped the health delivery system would make it easier for them to access needed services. Nine TB patients expressed the hope that medicines could be delivered to patients’ homes or to collection points nearer to their homes than the PHC clinics. One HIV patient requested that healthcare providers provide a faster service.

g. Factors underlying the patient satisfaction data

We retained five factors in factor analysis of the patient satisfaction data for both HIV and TB patients with eigenvalues > 1.00. The five factors accounted for 58% of the variance in satisfaction for HIV patients and 60% for TB patients. Table 4 shows the factor loadings for each of the variables. Based on the factor loadings, we labeled the five factors underlying the HIV data and the TB data (availability, accommodation, acceptability, and communication for both HIV and TB, and health worker preference for HIV and global satisfaction for TB). The labels capture the content of the different variables that load heavily on the individual factors.

Generally, patient demographic characteristics were not significantly associated with the satisfaction factors in multivariable analysis for both HIV and TB patients (Table 5). However, male HIV patients were less likely to be satisfied with the availability of resources than females, while among TB patients male patients and patients who had ever been married were less satisfied than female patients or patients who had never been married with the degree to which the health systems structures and processes accommodated their demands. TB patients with secondary and higher level of education were more likely to be satisfied with the quality of communication than those with no education.

**Table 2:** Socio-demographic characteristics of HIV (N = 300) and TB (N = 296) patients

**Variable**		**Types of care**		**p-value**
		**HIV**	**TB**	
Sex	Female	186 (62%)	156 (53%)	p = 0.03
Age (years)*		Mean (40) 95% CI (39-41) Median (39)	Mean (38) 95% CI (37-39) Median (37)	p = 0.07
		range (21-89)	range (18-82)	
Less than 20-29†		45 (15%)	73 (25%)	p = 0.04
30-39		113 (38%)	102 (34%)	
40-49		91 (31%)	77 (26%)	
> = 50		49 (16%)	44 (15%)	
Education				p = 0.43
None		55 (18%)	50 (17%)	
Primary		104 (35%)	92 (31%)	
Secondary		101 (34%)	101 (34%)	
Matric‡ and higher		40 (13%)	53 (18%)	
Employed*	Yes	36 (12%)	28 (9%)	p = 0.36
Marital status*§	Never married	238 (80%)	249 (84%)	p = 0.15

*Information on age was missing two HIV patients and marital status was missing in one HIV patient. Information on employment status was missing in one TB patient.

†12 people receiving TB treatment and care were less than 20 years old.

‡Matric is the final year of high school in South Africa.

§Ever married: currently married, divorced or separated, widowed.

The p-values indicate the significance of the difference between values among HIV and TB patients.

**Table 3:** Indicators of reported satisfaction of patients utilizing HIV (N = 300) and TB (N = 296) treatment

**Variable**	**Types of care**		
	**HIV**	**TB**	**p-value**
**Global satisfaction**	n = 293	n = 296	
How satisfied were you with the service today?			
Very satisfied/satisfied	278 (95%)	286 (97%)	p = 0.31
**Staff-patient communication**	n = 300	n = 294	
The doctors and nurses (health workers) discussed treatment fully with me			
Agree	300 (100%)	283 (96%)	p ≤ 0.001
	n = 226	n = 205	
I find it easy to tell the health worker when I have missed taking my tablets			
Agree	204 (90%)	194 (95%)	p = 0.09
	n = 300	n = 294	
It is a problem that health workers do not speak my language			
Agree	4 (1%)	4 (1%)	p = 0.63
	n = 288	n = 290	
The health workers are too busy to listen to my problems			
Agree	42 (15%)	43 (15%)	p = 0.93
**Staff attitudes**	n = 226	n = 191	
Some staff do not treat patients with sufficient respect			
Agree	118 (52%)	77 (40%)	p = 0.02
	n = 300	n = 294	
The health workers I see respect me			p = 0.15
Agree	279 (93%)	282 (96%)	
**Privacy and confidentiality**	n = 286	n = 295	
In this clinic are you able to talk to the doctors or nurses in private?			
Always	241 (84%)	262 (89%)	p = 0.12
	n = 262	n = 230	
Patient information is kept confidential in this clinic			p = 0.43
Agree	251 (96%)	217 (94%)	
**Staffing and amenities**	n = 300	n = 296	
For your ARV (TB) treatment what would you prefer:			p = 0.21
To see a nurse in a nearby clinic or	299 (100%)	291 (99%)	
To travel further to see doctor	1 (0%)	4 (1%)	
	n = 275	n = 276	
The facilities (including waiting area and toilets) are dirty			
Agree	58 (21%)	58 (21%)	p = 0.98
	n = 298	n = 288	
The queues to see a doctor or nurse are too long at this facility			
Agree	195 (65%)	115 (40%)	p ≤ 0.001
	n = 299	n = 296	
How do you think the service in this clinic could be improved?			
Shorter queues: Yes	170 (57%)	104 (35%)	p ≤ 0.001
More health workers: Yes	171 (57%)	132 (45%)	p ≤ 0.001
Cleaner facilities: Yes	65 (22%)	68 (23%)	p = 0.72
Better patient facilities (toilets, waiting room area): Yes	201 (67%)	161 (54%)	p ≤ 0.001

The p-values indicate the significance of the difference of values among HIV and TB patients.

**Table 4:** Factor loadings for indicator variables for assessing patient satisfaction for HIV (n = 265) and TB (n = 259) patients

**Variable**	**HIV**					**TB**				
	**1**	**2**	**3**	**4**	**5**	**1**	**2**	**3**	**4**	**5**
	**AV**	**AD**	**AC**	**CN**	**HW**	**AV**	**AD**	**AC**	**CN**	**GS**
How satisfied were you with the service today?	−0.14	−0.17	0.77	−0.02	0.09	−0.12	−0.02	0.03	−0.06	0.84
The doctors and nurses (health workers) discussed treatment fully with me‡	-	-	-	-	-	0.07	−0.01	0.01	0.54	0.51
It is a problem that health workers do not speak my language	0.02	−0.02	0.08	0.83	0.02	−0.09	−0.06	0.08	−0.77	0.12
The health workers are too busy to listen to my problems	−0.04	0.42	0.35	−0.47	0.09	−0.14	−0.18	0.47	0.42	−0.04
The health workers I see respect me	0.08	0.20	0.66	0.15	−0.15	−0.42	0.49	−0.03	0.13	−0.20
In this clinic are you able to talk to the doctors or nurses in private?	−0.04	0.48	0.09	0.34	0.14	−0.10	−0.21	0.76	−0.06	0.22
For your ARV (TB) treatment what would you prefer: to see a nurse in a nearby clinic or to travel further to see doctor	0.02	0.02	−0.00	0.01	0.94	0.09	−0.27	−0.75	0.02	0.16
The facilities (including waiting area and toilets) are dirty	−0.17	0.76	−0.10	−0.09	0.03	−0.21	−0.49	0.14	0.39	0.12
The queues to see a doctor or nurse are too long at this facility	−0.73	0.02	0.06	0.01	−0.12	−0.77	0.06	0.03	−0.13	0.21
*How do you think the service in this clinic could be improved?* Shorter queues	0.80	−0.24	0.03	0.06	0.08	0.84	0.06	−0.12	−0.05	−0.01
More health workers	0.72	−0.06	−0.13	−0.00	−0.24	0.69	0.33	−0.04	0.06	0.08
Cleaner facilities	0.16	−0.74	−0.01	0.00	0.04	0.40	0.65	−0.01	−0.16	−0.10
Better patient facilities (toilets, waiting room area)	0.46	−0.02	0.11	−0.21	0.10	0.06	0.74	0.05	0.13	0.12

*AV, *Availability; *AD, *Accommodation; *AC, *Acceptability; *CN, *Communication; *HW*, Health worker preference; *GS,* Global satisfaction.

‡For HIV patients, this variable was dropped from the factor analysis, because there was no variation in the variable (see Table 3).

**Table 5:** Factors associated with patient satisfaction for patients utilizing HIV (n = 265) and TB (n = 259) treatment

**Patient demographic characteristics**	HIV coefficient (95% CI) p-value*					TB coefficient (95% CI) p-value*				
	**1**	**2**	**3**	**4**	**5**	**1**	**2**	**3**	**4**	**5**
	**AV**	**AD**	**AC**	**CN**	**HW**	**AV**	**AD**	**AC**	**CN**	**GS**
Sex: Male	**−0.21**	0.13	0.05	0.05	0.10	−0.22	**−0.20**	−0.31	−0.06	−0.11
	(−0.35– -0.07)	(−0.41–0.67)	(−0.37–0.47)	(−0.25–0.35)	(−0.33–0.54)	(−0.60–0.15)	(−0.39– -0.00)	(−0.39–0.33)	(−0.32–0.20)	(−0.53–0.32)
	0.02	0.51	0.74	0.63	0.50	0.17	0.05	0.82	0.55	0.53
Age	0.01	0.01	0.01	−0.01	−0.02	−0.01	0.00	0.01	0.02	−0.01
	(−0.03–0.04)	(−0.03–0.46)	(−0.02–0.03)	(−0.03–0.02)	(−0.08–0.04)	(−0.03–0.02)	(-0.01–0.02)	(−0.00–0.02)	(−0.00–0.04)	(−0.02–0.01)
	0.56	0.49	0.53	0.47	0.45	0.36	0.53	0.06	0.10	0.28
Education: Primary	0.05	0.18	0.04	0.24	0.15	0.10	0.37	0.29	0.37	0.01
	(−0.57–0.67)	(−0.50–0.87)	(−0.16–0.25)	(−0.25–0.72)	(−0.12–0.43)	(−0.25–0.44)	(−0.23–0.97)	(−0.37–0.95)	(−0.07–0.82)	(−0.11–0.13)
	0.80	0.45	0.57	0.22	0.17	0.48	0.16	0.29	0.08	0.77
Secondary and higher	0.10	0.58	0.09	−0.09	−0.18	−0.12	0.02	0.22	**0.56**	−0.38
	(−0.47–0.67)	(−0.17–1.33)	(−0.19–0.36)	(−0.62–0.43)	(−1.06–0.70)	(−0.77–0.54)	(−0.86–0.90)	(−0.32–0.76)	(0.32–0.79)	(−0.90–0.14)
	0.62	0.09	0.39	0.62	0.57	0.64	0.96	0.32	0.00	0.11
Employed: Yes	0.27	−0.30	−0.08	0.20	−0.05	0.14	0.21	−0.17	0.06	0.22
	(−0.36–0.91)	(−1.01–0.40)	(−0.36–0.19)	(−0.97–1.36)	(−0.19–0.09)	(−0.34–0.62)	(−0.22–0.64)	(−1.19–0.85)	(−0.55–0.68)	(−0.36–0.79)
	0.27	0.26	0.41	0.63	0.33	0.46	0.25	0.67	0.79	0.35
Marital status: Ever married	−0.04	−0.03	0.10	−0.04	0.04	0.01	**−0.43**	−0.15	0.06	−0.08
	(−0.22–0.14)	(−8.88–0.83)	(−0.30–0.51)	(−0.22–0.13)	(−0.28–0.36)	(−0.36–0.38)	(−0.71– -0.14)	(−0.66–0.37)	(−0.17–0.2)	(−0.52–0.35)
	0.57	0.93	0.48	0.49	0.73	0.94	0.01	0.48	0.50	0.62

*Coefficients that are statistically significant at the 0.05 level are shown in bold font.

*AV, *Availability; *AD, *Accommodation; *AC, *Acceptability; *CN, *Communication; *HW*, Health worker preference; *GS,* Global satisfaction.

## Discussion

The subjective concept of patient satisfaction is an important intrinsic outcome of healthcare delivery, and it can instrumentally affect health outcomes because it determines long-term retention in care as well as adherence. In scaling-up HIV and TB treatment in sub-Saharan Africa, programme managers should thus not only focus on increasing the number of patients on treatment to decrease HIV-related mortality [9], but also on aspects of treatment delivery that could affect patient satisfaction.

Patients attending HIV and TB treatment services in this typical rural South African community reported high levels of overall satisfaction with their experience. However, as has been found in other studies [16,36,43,48], the high overall satisfaction level masked substantial dissatisfaction with particular aspects of the services, including the availability of health workers, the respect health workers showed patients, waiting times, and cleanliness of facilities.

HIV patients reported being less satisfied with some aspects of quality of care than TB patients (in particular, respectfulness of treatment, waiting times, and availability of waiting areas and toilets). These differentials in satisfaction levels between HIV and TB patients are likely due to historical differences in the organizations of healthcare delivery – the HIV treatment programme is much younger and still learning how to best organize service delivery – and differences in the speed of increase of patient load – unlike the TB treatment programme, the HIV treatment programme experienced an extremely rapid increase in patient load, which is likely to have led to temporary mismatches between human and physical resources for service delivery and patient demands. However, most satisfaction indicators were similar in HIV and TB patients except for two indicators, suggesting that in general treatment structures and processes do not differ significantly across the two programmes.

The HIV and TB programmes in the study area are supposed to follow the national guidelines for HIV and TB treatment and care, which are intended to be appropriate for nurse-led treatment and lay-out in detail which aspects of treatment should be discussed with patients [49,50]. Our findings that almost all patients reported that treatment was discussed fully with them and that the nurse-based care was highly acceptable can thus be interpreted as an indicator that the nurse-led and guideline-based HIV and TB treatment strategies are successful. However, healthcare providers may sometimes feel pressured to see many patients in a short space of time because of the high patient load leading to concerns by some patients that the health workers were too busy to listen to their problems. A study in Ethiopia found poor staff communication skills and lack of empathy to be factors affecting patient satisfaction [51]. Patients’ ability to freely talk about missed doses or their problems with their healthcare provider is important for improving treatment outcomes and adherence which are essential for the full treatment benefits for both HIV and TB to be realized [52,53].

Whereas overall relatively large proportions of HIV (52%) and TB (40%) patients reported that some healthcare staff did not treat patients with sufficient respect, the vast majority of patients in both groups (HIV 93%, TB 96%) agreed that they were personally treated with respect by the health worker who attended to them. This could be an indicator that patients have a higher tolerability for treatment lacking respect in their own encounters with health workers rather than in observed encounters of other patients. It is also plausible that patients wrongly report that they have been treated respectfully because of fear of negative consequences when complaining about their own health workers or because they feel such an answer is generally socially desirable. In developed countries, perception about staff respect has been found to be related to race and language, with minority groups reporting highest levels of disrespect [54]. In our setting all participants and health workers were from the same race and shared the same primary language (*isiZulu*). Future studies need to explore in more detail how health workers communication skills and attitudes can be improved to ensure that patients feel respected and understood in this community.

Both HIV and TB patients reported they were not able to always speak to healthcare providers in private. Privacy and confidentiality have been found to be strong predictors of patient satisfaction when seeking and utilizing care [55]. Patients need to be treated in private and their information should be seen to be kept confidential, so that they continue utilizing care. This is especially relevant in our study area where most patients received treatment from the clinic that was nearest to their homes [56-58]. Patient lack of trust with their healthcare provider has negative effects on patient satisfaction, treatment adherence and ultimately improved health status [59].

As in our study, several previous studies have found waiting times due to queues to be a main determinant of patient satisfaction [36,41,60]. HIV patients were significantly more dissatisfied with the length of the queues than TB patients. Indeed, based on observation and practice in both programmes, it is clear that queuing times for TB treatment are usually shorter than for HIV patients. This difference arises because TB patients join one queue to collect their treatment; the data clerk and TB nurse are in the same room to provide the patient clinic file and offer counselling before giving out treatment. In contrast, HIV patients normally have to join two queues -- first to see a counsellor and then to see a nurse for clinical assessment and medication. HIV patient queues are even longer on days when the doctor visits the clinics for patient examination and initiation of patients on ART – this was before nurse-initiated ART was introduced in 2011. At present, ART initiation does not happen on specific days when the doctor is available but it can happen on any day. Additionally, there are generally more patients on ART than TB treatment in the study area. Patients offered interesting suggestions to improve the queuing system -- health workers should work in shifts and that doctors should always be available at the clinics – and to make queuing a more pleasant experience – by providing food rations, and to reduce travel times to and from the clinics – by providing transport.

Patient satisfaction is the perceived fulfillment of patients’ needs and desires through the delivery of healthcare. As such, it will depend not only on the objective quality of care provided but also on patients’ expectations [15,51]. These expectations are known to vary with patient socio-demographic characteristics, with time and by context. Some studies of patient satisfaction thus attempt to ‘anchor’ the patient evaluations through the use of ‘anchoring vignettes’, i.e., short descriptions of experiences of other patients in utilizing healthcare, which participants in patient satisfaction surveys are asked to evaluate. Such anchoring approaches will provide an important improvement in our ability to compare patient satisfaction in this setting compared to other settings in the Southern African region and globally. However, for the comparison in satisfaction between HIV and TB patients in this study, it is unlikely that anchoring of patient responses would have substantially changed our findings, since HIV and TB treatment are delivered in very similar contexts, in close proximity to each other and within the same general PHC clinics.

Five factors were found to be underlying both the HIV and the TB patient satisfaction data. Four of these factors – which captured availability, accommodation, and acceptability of services, and the quality of communication – were similar in their representation of specific variables in the HIV and TB programme, pointing towards general constructs of patient satisfaction rather than disease-specific constructs. It is interesting to note that three of these underlying factors resemble closely three of the five dimensions of healthcare access identified by Penchansky and Thomas (1981) in their conception of access as the degree of fit between the health system and patient needs and wants [61]. In as far as patient satisfaction reports determine access; our findings thus partially validate this conception. A relationship between patient satisfaction and healthcare utilization is likely to arise – patient satisfaction will determine future access; we expect more highly satisfied patients to be more likely to utilize treatment in the same clinic again. Furthermore, patients can share their experience with others, which in turn can influence their access to care when the need arises. The different underlying factors were regarding global satisfaction for TB patients and health worker preference for HIV patients. This finding could possibly indicate that although these patients utilize care at PHC clinics integrated at facility level; experiences, expectations and quality of care needs for HIV and TB patients may not be identical but may vary by the type of healthcare a patient is utilizing -- the issue of health worker preference is more crucial for HIV patients probably because of the nature of the disease and its demands in healthcare provision.

Some studies have found that patient characteristics such as age and sex influence patient satisfaction probably because of lower expectations of healthcare and reluctance to articulate their dissatisfaction particularly among men and older patients [16,42]. In some studies in sub-Saharan Africa, patients with higher education were less satisfied with the level of privacy received at public sector HIV services [60], while women reported low levels of confidentiality with patient HIV test results [41]. However, in this study patient characteristics generally did not significantly influence patient satisfaction, indicating that the health systems structures and processes affected all patients roughly equally. However, we found that men receiving ART were more likely to complain about availability of services than women, possibly because they are more likely to work in the formal sector, where absenteeism is more likely to have negative consequences than in the informal sector and home production. We also found that those with a higher level of education were more likely to be satisfied in general with the level of health worker communication compared to those with no education. Patients with a higher level of education are likely to express greater dissatisfaction with the service received because they are more assertive and more aware of their patient rights and information needs than less educated patients.

## Conclusions

HIV and TB patients’ evaluations of specific healthcare delivery aspects revealed substantial dissatisfaction hidden in the global assessment of satisfaction. A wide range of patient satisfaction variables could be reduced to a few underlying factors that align broadly with concepts previously identified in the literature as affecting access to healthcare. Although patients reported high levels of general patient satisfaction, dissatisfaction with specific dimensions of care – in particular, health worker respect, queuing times, and availability and cleanliness of facilities – point towards possible interventions to improve patient satisfaction. Such improvements will be critical to maintain and further improve the performance of both the HIV and the TB programme in this typical rural South African community.

## Competing interests

The authors declare that they have no competing interests.

## Authors’ contributions

The authors NC, MLN and TB contributed to the conception and design of the article and revising it critically. NC did the acquisition of data and drafted the article, NC and TB did the analysis and interpretation of data. All authors have read and approved the final manuscript.

## Pre-publication history

The pre-publication history for this paper can be accessed here:

http://www.biomedcentral.com/1472-6963/14/32/prepub
